# Probing the Kinetic Stabilities of Friedreich’s Ataxia Clinical Variants Using a Solid Phase GroEL Chaperonin Capture Platform

**DOI:** 10.3390/biom4040956

**Published:** 2014-10-20

**Authors:** Ana R. Correia, Subhashchandra Naik, Mark T. Fisher, Cláudio M. Gomes

**Affiliations:** 1 Instituto de Tecnologia Química e Biológica António Xavier, Universidade Nova de Lisboa, Av. da República, EAN, Oeiras 2784-505, Portugal; E-Mail: acorreia@caltech.edu; 2 Department of Biochemistry and Molecular Biology, Hemenway Life Sciences Innovation Center (HLSIC), University of Kansas Medical Center, 3901 Rainbow Blvd., Kansas City, KS 66160, USA; E-Mail: snaik@udel.edu

**Keywords:** clinical mutants, missense mutants, frataxin, Friedreich’s ataxia, protein folding, chaperonin, osmolytes, protein stability, protein misfolding and misfolding diseases

## Abstract

Numerous human diseases are caused by protein folding defects where the protein may become more susceptible to degradation or aggregation. Aberrant protein folding can affect the kinetic stability of the proteins even if these proteins appear to be soluble *in vivo*. Experimental discrimination between functional properly folded and misfolded nonfunctional conformers is not always straightforward at near physiological conditions. The differences in the kinetic behavior of two initially folded frataxin clinical variants were examined using a high affinity chaperonin kinetic trap approach at 25 °C. The kinetically stable wild type frataxin (FXN) shows no visible partitioning onto the chaperonin. In contrast, the clinical variants FXN-p.Asp122Tyr and FXN-p.Ile154Phe kinetically populate partial folded forms that tightly bind the GroEL chaperonin platform. The initially soluble FXN-p.Ile154Phe variant partitions onto GroEL more rapidly and is more kinetically liable. These differences in kinetic stability were confirmed using differential scanning fluorimetry. The kinetic and aggregation stability differences of these variants may lead to the distinct functional impairments described in Friedreich’s ataxia, the neurodegenerative disease associated to frataxin functional deficiency. This chaperonin platform approach may be useful for identifying small molecule stabilizers since stabilizing ligands to frataxin variants should lead to a concomitant decrease in chaperonin binding.

## 1. Introduction

Many protein folding diseases result from missense mutants and are characterized by aberrant protein folding kinetics, leading to diminished steady state populations of the properly folded forms in the cell. Although some missense mutants can still acquire the correct fold under permissive conditions, they can be detrimental to the organism because: (1) they are susceptible to rapid degradation; (2) they result in lower steady state populations due to increased misfolding during synthesis and/or (3) they are more susceptible to denaturation during mild thermal stress (fever or heat stress). These mutants may be soluble initially after synthesis but subsequent kinetic partitioning onto the intracellular chaperone proteostatic machinery can lead to added stress due to increased maintenance of the correct fold. Alternatively, the mutants may partition onto the proteostatic machinery, shunting the protein toward proteolysis which results in an overall steady state decrease and loss of functional protein [1].

Over the past two decades there has been an increased interest in finding corrective strategies to ameliorate aberrant folds through stabilization of the native fold, manifested through the specific binding of small molecule pharmacological chaperones [2,3,4,5,6]. This strategy results in favorable shifts in the unfolding/folding equilibria toward the correctly folded forms. These efforts have ranged from controlling protein levels, inducing the heat shock response or developing small molecule protein stabilizer therapies (reviewed, e.g., in [7]). In some instances, combinations of some of these strategies are synergistic [5,8,9].

Frataxin is a small mitochondrial protein whose reduced functional levels are associated with the development of Friedreich’s ataxia (FRDA), a neurodegenerative condition characterized by progressive ataxia and cardiomyopathy. Most patients are homozygous for a GAA triplet expansion on frataxin gene which results in reduced transcription efficiency. Other patients are compound heterozygous, containing the triplet expansion in one allele and a deleterious point mutation on the other. Here, we have been particularly interested in distinguishing the biophysical differences between two missense FRDA mutants that show very similar thermal and chemical equilibrium stabilities [10,11] but result in quite distinct disease phenotypes [12,13]. While FXN-p.Asp122Tyr is associated with a mild phenotype in GAA/FXN-p.Asp122Tyr, heterozygotic individuals containing FXN-p.Ile154Phe manifest a more severe disease phenotype. It is still unclear how and where the folding defects of these frataxin mutants are manifested (*i.e.*, by poor protein synthesis, by protein misfolding inside/outside the mitochondria that is either triggered by or enhanced by different metal concentrations [14], variations in oxidative stress [15,16], *etc.*). In this instance, heterozygous FRDA can be considered to represent protein misfolding disease conditions and thus it may be beneficial to develop strategies to specifically increase the steady state stability of the initially soluble missense folding mutants using small molecule chemical chaperone approaches. Previous studies elicited dramatically different folding efficiencies between these FXN variants upon bacterial expression. While approximately 50% of FXN-p.Asp122Tyr is expressed in the soluble form, FXN-p.Ile154Phe is mostly (~80%) expressed as insoluble aggregates [11]. To further explore and identify the difference between these two variants, we have looked into their kinetic stabilities starting from purified variants. One normally tests kinetic stabilities of proteins by examining differences in unfolding kinetics in denaturants; however, this approach examines the consequences of a global unfolding reaction within harsh denaturant conditions. Given these caveats, it is best to detect the kinetic stability of these transients at near-physiological conditions. Under these particular conditions, the unfolded protein population is kinetically accessible but is not the dominant form at equilibrium. The main goal of this work to determine if one can capture these transient dynamic states as they establish, using a kinetic/thermodynamic partitioning/trap, the high-affinity nucleotide-free form of the chaperonin GroEL [6,17,18]. Studies with other protein substrates using this chaperonin capture species platform indicate that the binding affinities of transient partially folded proteins for GroEL can be quite strong, which should result in an observable kinetic partitioning binding profile [11,18,19,20,21]. Additionally, the ability of strong and weak folding osmolytes to: (1) increase *in vivo* solubility; (2) increase kinetic thermal stability and (3) diminish the partitioning of unfolded protein onto the chaperonin GroEL *in vitro* was examined. Curiously, it was found that the FXN-p.Ile154Phe mutant has the tendency to aggregate in the presence of a strong folding osmolyte such as TriMethylAmine N-oxide (TMAO). The kinetic differences observed for these frataxin mutant variants may correlate with the severity of Friedreich’s ataxia prognosis. Ultimately, developing easy-to-use platforms to detect and reverse the kinetic liability of these particular mutants may be a reasonable focal point for developing therapeutic strategies to increase the population of correctly folded frataxin monomers.

## 2. Results and Discussion

### 2.1. Establishment of Kinetic Partitioning Conditions

The GroEL chaperonin kinetic capture method allows one to detect transient partially folded/unfolded species which are in a dynamic equilibrium with native folds. This partitioning occurs because the transiently populated conformers are kinetically captured by the high affinity GroEL (sub-nanomolar K_d_s) [20,21]), resulting in a gradual decrease in unbound protein [19,20,22,23]. The equilibrium dynamics and partitioning onto GroEL can be further accelerated by adding non-denaturing concentrations of chemical denaturants (e.g., 1 M urea), slightly elevated temperatures (e.g., 40–45 °C) or both. These conditions accelerate the unfolding ↔ refolding transitions and allow one to perform the partitioning experiments in more reasonable time frames [20]. The above mentioned conditions are innocuous to GroEL stability and function, as this chaperonin remains in its stable tetradecamer protein binding competent state under over a wide range of temperatures (20–65 °C) in the presence of mild denaturant concentrations (1 M urea) [24,25,26].

**Figure 1:**
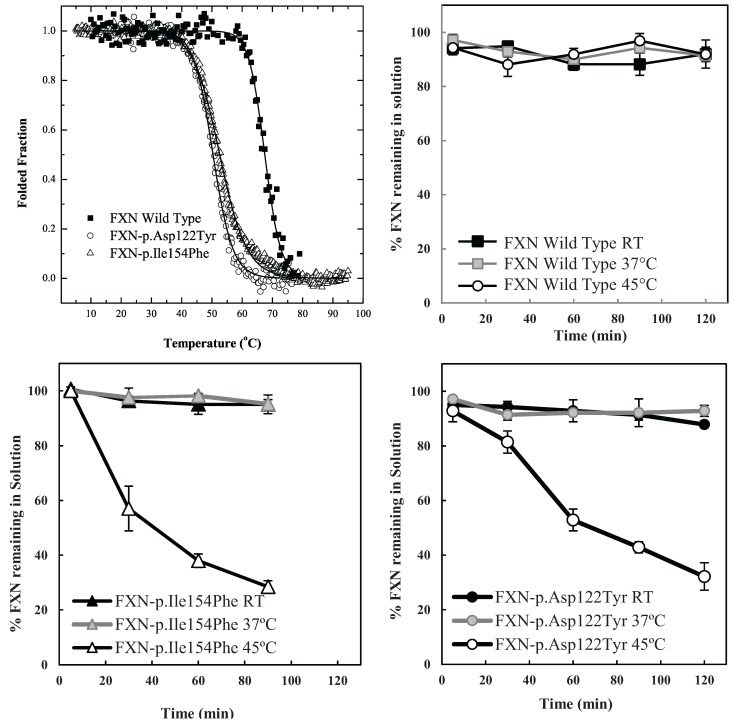
Comparison between the stability profile of wild type frataxin (FXN) and two clinical mutants FXN-p.Asp122Tyr and FXN-p.Ile154Phe. (**A**) Thermal denaturation curves of (■) FXN (Tm = 66.3 ± 0.1 °C), (○) FXN-p.Asp122Tyr (Tm = 50.4 ± 0.1 °C) and (Δ) FXN-p.Ile154Phe (Tm = 50.7 ± 0.1 °C) (Curves redrawn from [11] to highlight variants used herein) demonstrate differences between wt FXN and the two mutants; (**B**–**D**) Effect of temperature on Frataxin partitioning profiles (starting concentration 2 µM Frataxin) onto 2 µM immobilized GroEL oligomer beads in the presence of 1 M Urea. The partitioning of (**B**) Wild type FXN, (**C**) FXN-p.Asp122Tyr (circles,) and (**D**) FXN-p.Ile154Phe (triangles) was monitored by UV-visible spectroscopy at 25 °C, 37 °C and 45 °C to demonstrate that stability differences observed in (**A**) can be recapitulated with the GroEL chaperonin sink assay.

The particular conditions chosen to follow the kinetic partitioning of frataxin missense mutants (FXN-p.Asp122Tyr and FXN-p.Ile154Phe) were derived from examining thermal denaturation scan profiles (Figure 1A) [11]. According to these thermal profiles, it was hypothesized that at ~40 °C and above, both frataxin variants will begin to populate a steady state concentration of non-native forms whereas the Wild Type (wt) FXN should remain primarily in its native folded form up to ~65 °C. The partitioning kinetics of the different variants onto the high affinity form GroEL in the presence of 1 M urea were initially assessed at temperatures of 25 °C, 37 °C and 45 °C. At this urea concentration, none of the protein variants appear to readily populate denatured forms at 25 °C [11]. The remaining concentration of the folded soluble native proteins that have not partitioned onto the chaperonin bead platform (which is easily removed) can be easily followed by measuring the time dependent changes in the UV-visible absorbance of the soluble proteins at Abs 280–Abs 350 nm (to compensate for slight baseline shifts) or by examining the amount of protein remaining in solution using SDS PAGE analysis (Figure 1 and Supplementary Figure S1). The integrity of the remaining soluble protein is also easily deduced by monitoring the increased light scattering using UV-visible spectroscopy that occurs when a soluble protein undergoes slow aggregation (an upward shift in absorbance baseline from 350–220 nm). The native wild type frataxin shows no time dependent decline in the amount of total soluble protein under these slight denaturing conditions (1 M urea, 25–45 °C), and no partitioning onto GroEL could be detected (Figure 1B). Likewise, neither of the frataxin variants partitioned onto GroEL at 25 °C or 37 °C in the presence of 1 M urea, indicating that, at these temperatures, the most prevalent population of both variants remain in their predominantly folded soluble states. It is only as the temperature is increased to 45 °C that the transient unfolded/partially folded forms show significant partitioning and capture by the immobilized GroEL beads (Figure 1C,D). The initial test partitioning experiments (Figure 1B–D) used an estimated 1:1 molar ratio of GroEL (oligomer) to frataxin monomer (See methods for details describing the estimation of immobilized GroEL concentrations). The prediction that arises from these initial partitioning experiments states that simply increasing the immobilized chaperonin bead concentration should increase the partitioning rate of these frataxin missense mutants onto GroEL.

### 2.2. Initial Partitioning Rate Profiles are Dependent on the Solid Phase GroEL Concentration

**Figure 2:**
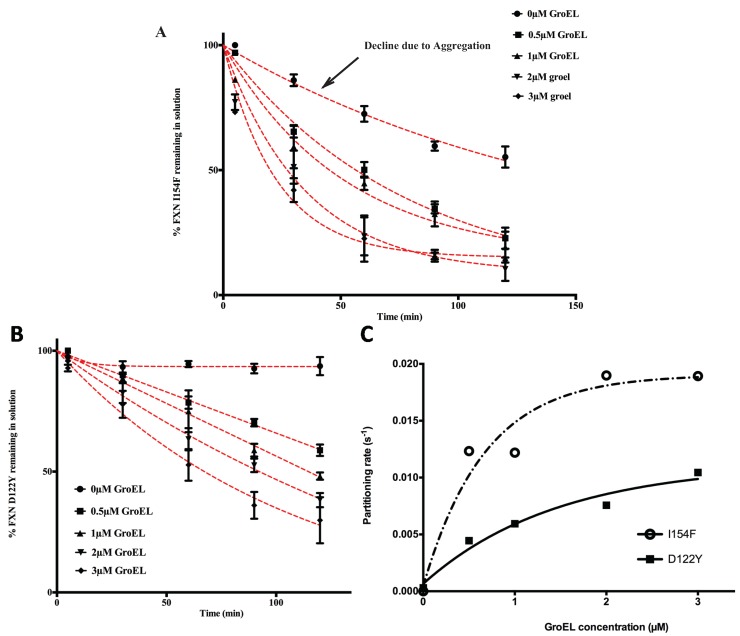
Effect of GroEL concentration on frataxin clinical variants partitioning profiles at 45 °C, 1 M urea. Frataxin Partitioning (2 µM) was analyzed using different estimated GroEL oligomer concentrations: control-no GroEL (●), 0.5 µM (■), 1 µM (▲), 2 µM (▼) and 3 µM (♦); (**A**) FXN-p.Ile154Phe (FXN I154F) and (**B**) FXN-p.Asp122Tyr (FXN D122Y). Each time point consisted of three separate spectroscopic measurements and the error bars represent ± 1 S.D. The pseudo first order fits to the data are represented by the red dotted lines. The values for each fit are listed in Supplementary Table S1; (**C**) The individual time point data representing the remaining frataxin in solution for each GroEL concentration series were fit to a pseudo first order kinetic profile. The pseudo first order partitioning rate increases as the GroEL concentration increases. The GroEL concentration is in excess of the amount of the partially folded frataxin concentration (not the total frataxin concentration). The remaining FXN concentrations declining with time, *i.e.*, the partitioning curves (in **A** and **B**) were fit to a pseudo-first order relationship to obtain the partitioning rates. A plot of the macroscopic partitioning pseudo first order rate *vs.* estimated GroEL concentration follows a hyperbolic relationship that tends towards saturation at higher GroEL concentrations.

Based on previous kinetic observations [20,27] the interaction between GroEL and dynamic partially folded transient populations is dictated by the unfolding rates of the natively folded protein to folding intermediate transitions. As the GroEL concentration increases, the apparent partitioning/GroEL kinetic capture increases through mass action effects. Since we are only examining the partitioning of the protein onto GroEL by measuring the remaining protein in solution, one should observe the following trend. As the GroEL concentration increases, the measured partitioning rates should approach a limiting value because these rates should only reflect the unfolding transition rates that are independent of the GroEL concentration [19,27]. Equation (1) resembles the equilibrium reaction previously described by Clark and Frieden [27]. Unlike the extensive and well described rate constant measurements and kinetic modeling in the Frieden work, our kinetic methodology does not examine the intrinsic microscopic unfolding rates directly but only examines the overall apparent macroscopic partitioning rates onto GroEL due to mass action effects. Over the short time span of the assay, the slow partitioning of the variants onto GroEL most likely indicates that the capture of the dynamic population of the transient unfolded/partially folded species is small compared with that of the native population. Even at low concentrations, the chaperonin concentration should be in vast excess compared with these transient and dynamic misfolded/unfolded mutant frataxin populations. The concentration of GroEL capture sites exceeds the oligomer concentration since GroEL has two binding sites that can efficiently capture small proteins. Indeed, Clark and Frieden demonstrated that the stoichiometry of partitioning a dynamic dihydrofolate reductase monomer onto GroEL increases from 1 to at least 2 DHFR monomers per 1 GroEL oligomer at elevated temperatures [27]. From a kinetic perspective, this provides a reasonable explanation for the observation that the macroscopic partitioning kinetic profile (loss of frataxin in solution) typically fits a pseudo first order decay rate [11,18,19]. In addition, as was previously observed for rhodanese partitioning onto soluble GroEL [18], the observed partitioning rates for both transient frataxin variants onto the immobilized GroEL increase and then approach limiting rates as the GroEL concentration (on beads) increases (Figure 2A,B). Of note, Clark and Frieden also document that the kinetic amplitudes and apparent rates approach maximum limiting values as the GroEL concentration increases.

The partitioning profiles of the FXN-p.Ile154Phe frataxin mutant saturate at a higher limiting rate than does the FXN-p.Asp122Tyr. Likewise, the FXN-p.Ile154Phe partitioning rates are clearly faster than those observed for FXN-p.Asp122Tyr and show larger amplitudes of change (*i.e.*, more pronounced decline in observed soluble frataxin variants) as estimated GroEL bead concentrations increase (Figure 2). For instance, after a 60 min incubation at ~3 μM GroEL concentrations (see methods), the soluble fraction of FXN-p.Ile154Phe declines to ~25% of its original starting concentration, while under the same conditions, the amount of soluble FXN-p.Asp122Tyr is ~55% of its original concentration (Figure 2A,B). Also, the initial decline in the FXN-p.Ile154Phe soluble fraction shows a much sharper decline from the initial zero time point. Since the earliest time point of measure is one minute, it appears that a substantial amount of FXN-p.Ile154Phe partitions onto the GroEL platform during the first minute compared with the smaller initial changes with the FXN-p.Asp122Tyr variant. This data indicates that the FXN-p.Ile154Phe mutant partitions onto GroEL more rapidly than the FXN-p.Asp122Tyr mutant. These kinetic partitioning differences would have profound effects on protein steady state levels as these newly synthesized mutant variants encounter the chaperone surveillance network.

The macroscopic partitioning/binding kinetic rates were obtained by fitting the partitioning curves (decrease in percentage of soluble FXN Figure 2A,B) to a pseudo first order decay equation. To determine the binding characteristics, these rates (s^−1^) (listed in Supplementary Table S1) were plotted as a function of increasing GroEL concentration (Figure 2C). As predicted, the kinetics rates are non-linear with respect to the GroEL concentration and these rates appear to approach a limiting rate as the GroEL concentration increases [27]. Although the kinetic profile of the FXN-p.Asp122Tyr was not complete, the partitioning rates were assumed to represent a pseudo-first order decay. Certainly, the FXN-p.Ile154Phe shows a near complete kinetic partitioning profile as the GroEL bead concentration increases. This follows the other numerous instances where partitioning rate data of other proteins were also reasonably fit to pseudo first order decay profiles [19,20,21,27]. Our modified approach indicates that the partitioning kinetic rates of the substrate protein DHFR used by Clark and Frieden also approaches a limiting rate as the concentration of the immobilized GroEL bead system increases (Supplementary Figure S2).

**Figure 3:**
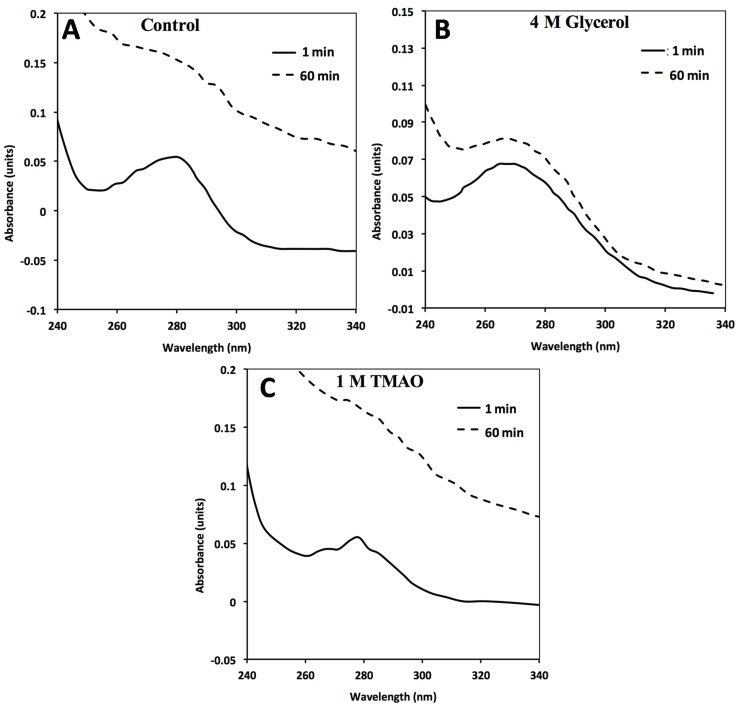
In the absence of GroEL beads, general FXN-p.Ile154Phe aggregation (2 µM) occurs in solution alone (no osmolytes) (**A**) in the presence of 4M Glycerol (**B**) or 1 M TMAO (**C**). FXN-p.Ile154Phe was incubated at 45 °C for 60 min in the absence and presence of the different osmolytes and the UV absorbance spectra at time 0 min and 60 min are represented. The spectra in the presence of 1 M TMAO shows a larger increase in light scattering contributions as assessed by the increase in the general baseline from general protein aggregation compared without osmolytes (**A**) or with glycerol (**B**). The 1 and 60 min spectra of FXN-p.Asp122Tyr under all three above conditions do not show a significant wavelength dependent shift in the baseline due to light scattering from aggregation (Supplementary Figure S3).

The behavior of the two mutants in solution in the absence of the GroEL capture platform also shows slight differences because the amount of recoverable FXN-p.Ile154Phe slowly declines in concentration even in the absence of the GroEL chaperonin (Figure 2A, control experiments). This slow decline is not detected in the FXN-p.Asp122Tyr control profile (Figure 2B). Spectroscopic analysis (*i.e.*, light scattering contributions) indicates that, under the conditions of the assay, the FXN-p.Ile154Phe variant slowly aggregates in the absence of GroEL (Figure 3A). The extent of aggregation in this control assay is most certainly dictated by the original starting concentration of FXN-p.Ile154Phe. It is important to note however, that when GroEL is present, this general aggregation side reaction is suppressed since the remaining soluble FXN-p.Ile154Phe mutant within the supernatant shows no evidence of aggregation-dependent light scattering contributions (no absorbance increases at 350 nm), particularly at the start of the partitioning reaction. This observation is completely in line with the well-established property that GroEL prevents large scale protein aggregation [22,28,29]. In the end-point analysis, it is clear that the FXN-p.Ile154Phe preferentially partitions onto GroEL at substantially higher limiting rates than does FXN-p.Asp122Tyr (Figure 2C).

In every case where dynamic transiently unfolded populations are in equilibrium with folded states, partitioning rates of the misfolded/partially folded population onto GroEL approach an apparent limiting plateau value, as the chaperonin concentration increases ([6,24], Figure 2C and Supplementary Figure S2). This observation now appears to be observed when soluble or immobilized GroEL capture platforms are employed. For the frataxin mutants tested here, this limiting partitioning rate is higher for the FXN-p.Ile154Phe mutant than for the FXN-p.Asp122Tyr variant. A similar difference in macroscopic partitioning kinetics appears to be present with various transthyretin (TTR) mutants that manifest varying degrees of disease severity *i.e.*, (A25T > L55P) [6]. However, unlike the case with the frataxin variants, the GroEL concentration was kept constant so the GroEL concentration influences on the macroscopic TTR partitioning rates were not examined for these two TTR variant mutants.

The most logical explanation for this partitioning rate plateau with increasing GroEL capture concentration is that this rate depends on the limiting unfolding/partial unfolding rate of the metastable proteins. At high GroEL concentrations, the unfolding/partial unfolding of the transient chaperonin binding conformer becomes rate limiting. Earlier experiments by Walter *et al.* [21] have demonstrated that the high affinity GroEL chaperonin does not actively unfold natively folded proteins nor does it affect the microscopic unfolding rate constants as GroEL concentrations increase. GroEL simply binds the partially folded populations as they form. Simple mass action effects shift the folding-unfolding equilibrium toward a GroEL-bound unfolded state [21,27,30].

### 2.3. Differential Scanning Fluorimetry (DSF) of Wild Type and Mutant Variants—Effect of Osmolytes

**Figure 4:**
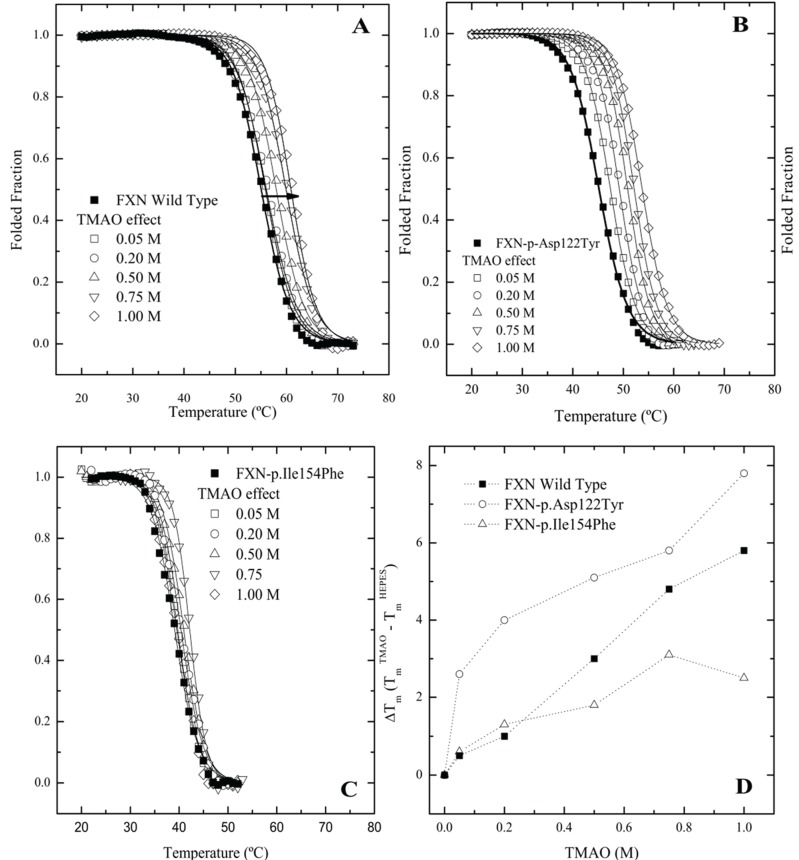
Differential Scanning fluorimetry (DSF) of native and variant frataxins in the absence and presence of TriMethylAmine N-oxide (TMAO). The concentration of frataxin was 4 µM. The dye Sypro orange was used to probe the temperature dependent protein unfolding reaction. The starting apparent kinetic T_m_ values without osmolyte were 55.1 °C for wild type FXN (**A**), 48.8 °C for FXN-p.Asp122Tyr (**B**) and 39.1 °C for FXN-p.Ile154Phe (**C**) (see also Table 1). Panels (**A–C**) Melting curves for the three variants, measured by Differential Scanning Fluorimetry in the presence of increasing concentrations of two osmolytes. Panel (**D**). TMAO efficiently increases the T_m_ values of all FXN variants in a concentration dependent way. (■) FXN wild type, (○) FXN-p.Asp122Tyr and (∆) FXN-p.Ile154Phe.

Since frataxin missense mutants show kinetic preferences for chaperonin partitioning, one would also predict that thermal kinetic denaturation profiles should also show a similar effect particularly in the presence of a binding fluorescent dye. In this instance, the presence of the dye binding to a transient partially folded species that is in rapid kinetic equilibrium with a native state may result in a dramatic shift in the kinetic T_m_ values toward lower temperatures. A series of kinetic differential scanning fluorimetry (DSF) measurements indicate that all the frataxin mutants show a substantial leftward shift in the kinetic melting temperature (T_m_) (Figure 4A–C No Osmolyte; Table 1, Control) compared with the equilibrium determined T_m_ values (Figure 1A). All the mutants show some stabilization in the presence of increasing TMAO concentrations with FXN-p.Ile154Phe showing the smallest shift in the kinetic T_m_ (Figure 4C, Table 1). This kinetic response is in line with the observed partitioning differences observed in the presence of the chaperonin platform (Figure 2A,B).

**Table 1:** Melting temperatures (*T*_m_) determined by the two state fits to the unfolding curves obtained by DSF experiments. The concentration of frataxin was 4 µM.

Control		Wild Type ^a^		FXN-D122Y ^a^		FXN-I154F ^a^	
		*T*_m_ (°C)	Δ*T*_m_	*T*_m_ (°C)	Δ*T*_m_	*T*_m_ (°C)	Δ*T*_m_
		55.1 ± 0.1	-	48.8 ± 0.1	-	39.1 ± 0.1	-
Trehalose (M)	0.025	54.6 ± 0.1	−0.5	50.1 ± 0.1	3.9	39.6 ± 0.1	0.5
	0.050	54.9 ± 0.1	−0.2	50.7 ± 0.1	4.5	39.5 ± 0.1	0.4
	0.125	55.3 ± 0.1	0.2	50.3 ± 0.1	4.1	38.3 ± 0.1	−0.8
	0.250	56.5 ± 0.1	1.4	50.3 ± 0.1	4.1	39.6 ± 0.1	0.5
	0.500	57.8 ± 0.1	2.7	53.4 ± 0.1	7.2	40.3 ± 0.1	1.2
	0.750	60.2 ± 0.1	5.1	54.1 ± 0.1	7.9	39.3 ± 0.2	0.2
TMAO (M)	0.05	50.1 ± 0.1	0.5	48.8 ± 0.1	2.6	39.7 ± 0.1	0.6
	0.2	51.3 ± 0.1	1.0	50.1 ± 0.1	4.0	38.4 ± 0.1	1.3
	0.5	52.0 ± 0.1	3.0	51.3 ± 0.1	5.1	40.9 ± 0.1	1.8
	0.75	54.0 ± 0.1	4.8	52.0 ± 0.1	5.8	42.2 ± 0.1	3.1
	1	48.8 ± 0.1	5.8	54.0 ± 0.1	7.8	41.6 ± 0.2	2.5

**^a^** The control kinetic *T*_m_ s are lower than the previously determined by CD or fluorescence spectroscopy and data indicates that precautions have to be implemented to avoid long incubations with dye before start starting the thermal scan. This artifact is typically induced by the presence of the dye binding to protein and shifting the equilibrium toward the unfolded form.

The DSF plots clearly indicate that the FXN-p.Ile154Phe mutant is less responsive to stabilization by a strong folding osmolyte such as TMAO, while the FXN-p.Asp122Tyr exhibits the most dramatic changes in the kinetic T_m_ values in the presence of different osmolytes. Additionally, it was observed that the FXN-p.Ile154Phe variant was particularly sensitive to the presence of the dye. In this instance, when the FXN-p.Ile154Phe was incubated for an extended period of time with the dye prior to starting the DSF experiment, the Sypro-Orange dye appears to induce protein unfolding and one could no longer detect the kinetic sigmoidal signal indicative of a transition from folded to unfolded forms. This might result from the conformational changes induced within the protein core by the Ile154 to Phe mutation. Previous data has shown that this variant is more flexible and is likely to have a more open conformation allowing the dye to bind to partially exposed hydrophobic patches on transient folding intermediates. This in turn may result in a kinetic shift of the folded population towards denaturation or aggregation [10,31]. Thus, special care was taken to minimize the time period that the dye was added to the protein prior to initiating the DSF experiments. This effect of the dye on protein unfolding/aggregation correlates with the effect these mutations have on the frataxin kinetic stability. Since various osmolytes can differentially stabilize transient folds, it is of interest to examine how the solubilities/GroEL partitioning properties/kinetic stabilities of these two mutants are affected by the presence of strong (TMAO) and weaker (polyol) folding osmolytes both *in vivo* and *in vitro*.

### 2.4. Assessing Mutant Frataxin Protein Solubility in Vivo and Examining in Vitro Partitioning onto the Solid Phase GroEL Platform in the Presence of Osmolyte Stabilizers

There is rising interest in developing small molecule stabilizer strategies to facilitate folding *in vivo* within bacterial systems, particularly for missense folding mutants that could serve as targets for pharmacological chaperone drug development. Novel engineered folding schemes by the Bardwell group have examined some test protein folding in the presence of external osmolytes to determine if folding could be enhanced in the periplasmic space with these additives [32]. Their findings indicated that the polyol osmolyte classes were particularly strong in enhancing *in vivo* folding while stronger folding osmolytes such as TMAO are sometimes not as effective perhaps due to the tendency of stronger osmolytes to induce aggregation (driving more rapidly to collapse). In other clever approaches, Gierash and colleagues showed that increasing intracellular concentrations of proline within bacterial systems avoided misfolding and decreased intracellular aggregation [33].

Researchers have also examined the possibility that simple osmolyte addition to growth media may enhance *in vivo* folding of mutant proteins within the bacterial expression system. A study by Kraus and colleagues specifically demonstrated that osmolyte addition leads to an increase in active functional cystathioine β synthase mutants [34]. Specifically, it is of interest to determine if adding folding osmolytes to *E. coli* growth media will shift the unfolded/partially unfolded populations of the frataxin missense mutants towards soluble, stable, correctly folded conformations *in vivo*. Since functional activity of frataxin is not easily measured, the stabilizing effects of adding these folding osmolytes to the frataxin clinical variant proteins were evaluated *in vitro* by examining their effects on chaperonin capture profiles.

**Figure 5:**
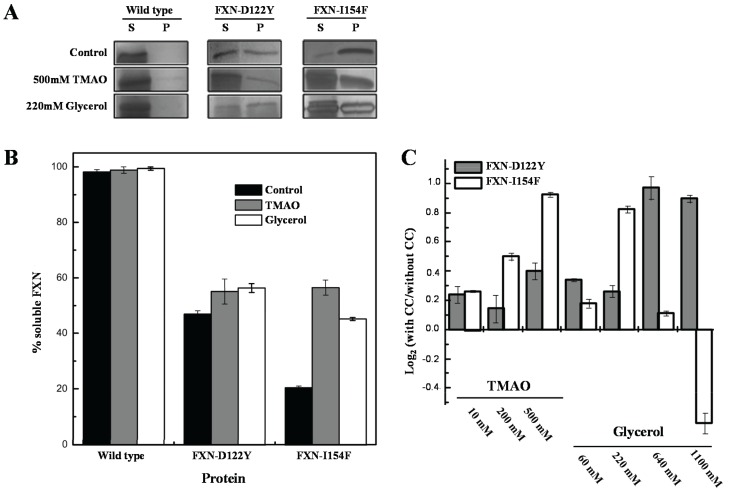
Effect of chemical chaperones on early folding events. (**A**) SDS/PAGE gels obtained from *E. coli* lysates expressing frataxin. For each protein variant, the soluble (S) and insoluble (P) fractions are shown; (**B**) Semi-quantitative analysis of the relative proportion of frataxin present in the soluble and insoluble fractions, obtained from densitometric analysis of gel bands (*n* = 3), allowed the determination of the protein expressed in the soluble form; (**C**) Variation of the folding efficiency induced by the presence of chemical chaperones (increasing concentrations of TMAO and glycerol). After the densitometric analysis of the gel bands, the ratio between the amount of soluble protein present in the presence and absence of the compounds were determined. The Log2 of this ratio is here shown to highlight the variation observed.

For the *in vivo* experiments, weak and strong folding osmolytes, such as TMAO and glycerol [35,36], were added to the growth media and protein solubilities were examined using standard solubility assays within cellular extracts (Figure 5). The solubility of the frataxin mutants showed increases (*i.e.*, remained in solution after removing insoluble material by centrifugation) with various concentrations of TMAO and a high concentration of the polyol glycerol. FXN-p.Asp122Tyr shows increases in solubility with both osmolytes while TMAO increases solubility for FXN-p.Ile154Phe and shows a decrease in solubility with lower concentrations of glycerol. The TMAO results are in relative agreement with the DSF experiments (Figure 4 and Table 1) although the smaller DSF shift in the kinetic T_m_ was expected to show a smaller increase in the amount of soluble frataxin mutants.

**Figure 6:**
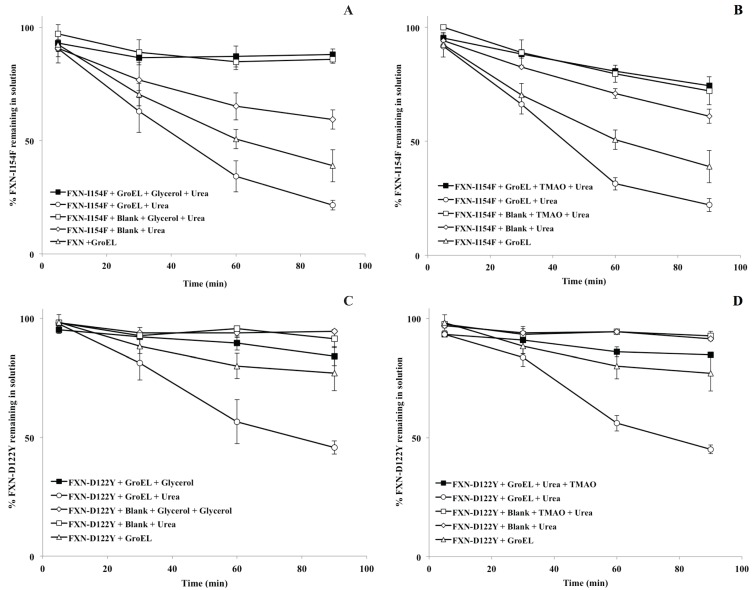
Partitioning profile changes of FXN-p.Ile154Phe (FXN-I154F) and FXN-p.Asp122Tyr (FXN-D122Y) (both at 2 µM) in the absence (solid triangle) and presence (solid box) of 2 µM GroEL beads in the presence of 4 M glycerol ((**A**,**C**), respectively) or 1 M TMAO ((**B**,**D**), respectively). In both instances, the non-denaturing concentration of urea is 1 M at 45 °C. (**A**,**C**). The osmolyte solubility/partitioning profiles were compared with time dependent solubility changes without osmolytes.

For the *in vitro* osmolyte experiments, the incubation of FXN-p.Ile154Phe and FXN-p.Asp122Tyr mutants with both 4 M glycerol and 1 M TMAO in the presence of the chaperonin resulted in a decline in the partitioning which is also in line with the solubility results (Figure 5A,B). Tentatively, the osmolytes appeared to induce stabilization (increase soluble fraction) of the native folds of FXN-p.Ile154Phe and FXN-p.Asp122Tyr. Both folding osmolytes (4 M Glycerol and 1 M TMAO) diminished the time dependent loss in apparent solubility compared with the profiles in the absence of osmolytes, regardless of the absence or presence of the GroEL capture platform (Figure 6). In contrast to the earlier gradual loss in small aggregates from brief differential centrifugation spin down (Figure 2A control), no apparent decline in the apparent soluble FXN-p.Ile154Phe was observed when glycerol and TMAO were present regardless of the presence of the GroEL bead capture platform (Figure 6A,B, open and closed squares). Since osmolytes, particularly TMAO, may induce rapid collapse and in some cases small aggregates, the UV-visible spectra of both mutants were compared at 1 min and 60 min incubation times at the beginning and the end of the time dependent incubation at 45 °C, 1 M urea (Figure 3A–C). Both TMAO and glycerol prevents partitioning of the FXN-p.Asp122Tyr mutant onto GroEL and very little increase in light scattering contributions (aggregation) were observed with this variant ruling out the possibility of large scale aggregation interference with GroEL partitioning (Supplementary Figure S3). In contrast, the addition of both osmolytes to the FXN-p.Ile154Phe mutant alone results in a clear aggregation dependent upward shift in the entire UV-visible spectral after a 60 min incubation (Figure 3A–C). The light scattering increase in the entire absorbance spectrum baseline is more pronounced for TMAO than it is for glycerol (Figure 3, compare panels C and B). This difference may be related to the tendency of a stronger folding osmolyte such as TMAO to easily induce aggregation rather than stabilize the correct fold [37,38]. It is worth noting that neither weak nor strong folding osmolytes prevent the binding of either completely denatured proteins or highly populated partially folded proteins onto GroEL [39,40]. The resulting aggregate size at 60 min was not measured in either case. Larger aggregate removal by centrifugation was clearly demonstrated in the absence of osmolytes or chaperones (see Figure 2A control). Alternatively, the removal of aggregates in the presence of osmolytes may be diminished due to viscosity differences although the temperature of the solution was elevated (45 °C) which reduces the solution viscosity. In either case, the addition of osmolytes to the FXN-p.Ile154Phe clearly shows an aggregation dependent increase in the absorbance spectra.

As with all methodologies, these experiments highlight some limitations and points to design alternatives to circumvent limitations of the method. The frataxin partitioning/stabilization experimental results indicate that solution protein aggregation reactions can interfere with the partitioning reaction (Figure 3), particularly if the aggregation collapse is rapid. Rapid aggregation interferes (competes) with the partitioning reaction of the protein onto GroEL, since chaperonins are unable to reverse protein aggregation once it occurs. The only exception to this observation comes from a situation where there is a reversible equilibrium between the large aggregate and monomeric capture-competent populations. In this instance, Apetri and Horwich showed that, under these specific conditions, the chaperonin could once again bind and trap these soluble transient folding populations that could once again undergo facilitated folding by the chaperonin system [41].

Rapid aggregate formation can decrease the time-dependent partitioning amplitudes, leading to a false positive result in identifying potential solution stabilizers. Also, although our test solutions are continually mixed with GroEL beads, the diffusion of the target protein (GroEL capture competent state) from the bulk solution to the GroEL immobilized on the bead surface decreases the collision and binding kinetics decreasing chaperonin capture efficiency compared with the situation where GroEL is in solution [19,20,27]. Even so, it was observed that elevating the concentration of the GroEL capture platform was sufficient to completely eliminate aggregation effects because the protein partitions onto the chaperone protein. Measured association constants between GroEL and partially folded or collapsed folding intermediates at 25 °C indicates that the kinetic range is anywhere from 10^8^ M^−1^ s^−1^ (diffusion controlled) [42] to 10^3^ M^−1^ s^−1^ [43]. These association rates varied due to the nature of the folding intermediate (degree of hydrophobicity), electrostatic nature of that intermediate (GroEL surface surrounding the binding site is negatively charged) and/or the lifetime or kinetic collapse of that particular intermediate [44]. Increasing the chaperonin concentration by using smaller bead constructs and effectively eliminating competing aggregation reactions will avoid false identification of stabilizers. It was previously shown that a large excess of soluble chaperonin (ratio 10 GroEL oligomers to 1 target protein molar ratio) effectively prevents competing aggregation side reactions, and under these conditions, all of the protein eventually partitions onto the chaperonin [19,21]. Nonetheless, it is quite clear that at high enough GroEL concentrations, one can use the high affinity GroEL chaperonin (no ATP) as a platform to readily identify protein-stabilizing conditions, even with aggregation-prone proteins like FXN-p.Ile154Phe frataxin or with previously observed results with aggregation-prone CFTR NBD1.

Strong and weak folding osmolytes are defined by the magnitude of free energy burial of the peptide backbone in proteins as defined by Bolen and colleagues [37,38], thus the different effects observed for each mutant may result from the different locations of the mutations within the protein structure. Ile154 is located within a fairly buried position within the protein core while Asp122 is located on a more solvent exposed loop region with crucial functional importance [45].

It is unclear at this time if the kinetic and aggregation differences that were observed for these two missense frataxin mutants could complicate the search for global therapeutic stabilizing ligands. For instance, it would be highly desirable to demonstrate that one small molecule ligand would globally stabilize a broad range of single site destabilization mutants within one protein. At this time, it is still premature to predict if stabilizing ligands/therapeutic compounds may be mutant specific for these clinical frataxin variants. The case for the existence of global ligand stabilizers is related to earlier demonstrations that showed that a multitude of site specific thermally destabilizing kinetic intermediates can be reversed by distant global site-specific suppressor mutations [46]. The goal of many in the protein misfolding disease field is to demonstrate and identify instances where global ligand binding would stabilize a wide range of missense folding mutants in a particular protein folding disease [45,47,48,49]. Otherwise, specific folding mutations may require specific stabilizers.

Our observations with solubility and chaperonin interactions agree with recent *in vivo* assessment indicating that solubility does not necessary correlate with increased function [1]. With the more aggregation prone FXN-p.Ile154Phe mutant, one can observe instances where an increase in *in vitro* aggregate signals does not result in the formation of aggregates that are large enough to be easily removed from solution using the standard centrifugation speeds used to spin down the chaperonin beads. Consequently, small aggregates that form within the cellular soluble extracts may also escape removal by common centrifugation practices. As shown here with the very stringent high-affinity nucleotide-free GroEL chaperonin capture system, small aggregates may be unable to interact with and partition onto many intracellular chaperones (referred to as holdases in [1]).

## 3. Experimental

Highly purified GroEL was obtained using a modified purification scheme [40]. NHS-derivatized Sepharose Fast Flow beads were purchased from GE Healthcare Inc. (Piscataway, NJ, USA). All other chemicals were of the highest purity available and purchased from Sigma (St. Louis, MO, USA).

### 3.1. Frataxin Protein Purification

All constructs were expressed in *Escherichia coli* competent cells (BL21 (DE3)) and the frataxin protein was purified as previously described [10,50,51]. Plasmid-derived protein expression was induced by addition of 0.5 mM IPTG (Sigma). As in previous studies, all protein variants contained the conserved C-terminal domain (amino acids 91–210). The vectors also contained a His tag at the C-terminal end of the protein to simplify purification [10]. The mutants were stable in solution at room temperature. The frataxin protein variants and native proteins were fast frozen and this procedure insured that all of the frataxin variants retained both their original spectroscopic properties and exhibited reproducible melting temperatures. Slow freezing was avoided since both purified mutants showed visible precipitation upon thawing when slow freezing procedures were performed.

### 3.2. Preparation and Validation of GroEL Beads

GroEL beads were prepared by incubating pure GroEL with activated NHS-Sepharose Fast Flow beads (GE Healthcare). The GroEL concentration on the beads was determined from the labeling efficiency and wet bead volumes. The wet bead volume was calculated by measuring the total void volume of the wet beads. Specifically, the wet beads were centrifuged to dryness in a spin column (Pierce Scientific, Rockford, IL, USA) and the collected buffer was indicative of the void volume. Measuring this void volume in conjunction with measuring the difference in the GroEL concentration prior to and after incubation with the activated NHS sepharose beads allowed us to calculate the concentration of GroEL bound to the beads. This concentration, however, is not an absolute value but an estimate of the protein immobilized on the beads. The beads were tested for their partitioning efficacy by measuring the partitioning and refolding of Dihydrofolate Reductase (DHFR) (Sigma). DHFR is transiently unfolded and is known to partition onto GroEL from its initial folded state. These interactions have been extensively documented [20,52]. Specifically, the DHFR test protein is incubated in the presence of GroEL at 37 °C for 60 min which results in a complete loss of observable activity due to capture by and partitioning onto the chaperonin. After this incubation time, the addition of 10 mM ATP (Sigma) and 4 M glycerol leads to substantial release and refolding of the chaperonin captured DHFR (>60% activity regain). The partitioning and refolding reactions are followed by measuring the activity of the non-partitioned protein or by following the decrease in protein absorbance. The DHFR activity is assayed by following the decrease in the UV absorbance of 7 µM NADPH at 340 nm in the presence of 5 µM Dihydrofolic Acid (Sigma) [51,52] (results shown in Supplementary Figure S2).

### 3.3. Solid Phase GroEL Partitioning Assay

Partitioning studies were carried out by incubating the wild type frataxin (FXN) and the mutants FXN-p.Asp122Tyr and FXN-p.Ile154Phe with the GroEL beads at 25 °C, 37 °C or 45 °C. 2 µM of wild type or variant frataxin was incubated with 2 µM GroEL beads (estimated) in buffer A (50 mM Tris, 50 mM KCl, 5 mM MgCl_2_, 0.5 mM EDTA, 1 mM DTT, pH 7.5) with 1 M urea and this reaction mixture was incubated with constant mixing on a rotary mixer at the described temperatures. GroEL remains tetradecameric and fully functional under these conditions [24,25]. Samples were withdrawn at the described time points and the soluble frataxin volume in the reaction mixture, which had not partitioned onto the GroEL beads, was quantified by UV absorbance spectroscopy (Shimadzu UV-2401, Shimadzu, Japan) at Abs 280 nm–Abs 350 nm (accounts for instrumental/system drift in baseline) or alternatively on a SDS/PAGE gel (Supplementary Figure S1). Once this absorbance time point was taken, the frataxin solution in the cuvette was reintroduced back into the assay in order to maintain a constant total frataxin protein concentration. Throughout the time course of the experiment, the volume of the sample was also measured at each time point (parafilm-sealed, closed microcentrifuge tubes) to ensure that this volume remained constant. No visible aggregate flocculants were observed for any of the frataxin samples. The entire absorbance spectrum was collected in each instance to detect any light scattering contributions due to aggregation.

### 3.4. Kinetic Analysis

#### 3.4.1. Frataxin

The typical binding constant of an unfolded protein with the nucleotide free GroEL chaperonin can approach those found in antibody-antigen interactions [36,53]. This capture/detection method kinetically and thermodynamically traps transient misfolded or partially folded populations, leading to a time dependent depletion in the total amount of soluble frataxin in solution, particularly when the chaperonin is engineered onto an immobilized bead support (simplified binding reaction below (Equation (1)). It is important to note that this simplified kinetic scheme only examines the depletion (end point analysis) of the soluble protein and does not include potential multiple species as was observed by Clay and Frieden [27,30,52].


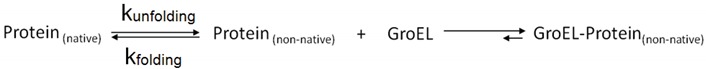
(1)

To measure the macroscopic kinetic partitioning rates associated with the GroEL-FXN mutant binding, each mutant (2 µM) was incubated with increasing estimated GroEL oligomer concentrations (0.5 µM–4 µM immobilized on beads) and the time dependent changes in concentration of frataxin remaining in solution was measured following the rapid removal of the GroEL beads (brief centrifugation at 1500× *g* for 30 s). At various time points, the amplitudes of the decline of soluble frataxin during partitioning reaction were recorded for each GroEL concentration. This time dependent decline in soluble protein amplitudes were fit to a first order decay curve using Prism curve fitting software (GraphPad Inc., La Jolla, CA, USA) to generate the partitioning rates at each GroEL concentration. These partitioning rates were then plotted as a function of GroEL concentration to obtain the partitioning curve for the FXN-p.Ile154Phe and FXN-p.Asp122Tyr frataxin mutants [18]. As was previously observed with GroEL dependent rhodanese partitioning kinetics [18,19], both the DHFR and frataxin variant proteins partition onto the chaperonin and the data were fit to pseudo first-order partitioning decay rates which then approach limiting values as the concentration of immobilized GroEL beads increases.

#### 3.4.2. DHFR

The kinetic partitioning rates for DHFR onto GroEL chaperonin attached beads were obtained in a manner similar to frataxin above with the following minor changes. A concentration of 1 µM of DHFR was incubated with increasing concentrations (0.5 µM–4 µM) of GroEL beads at 25 °C. Of interest, it was noted that even at the highest GroEL bead concentrations, DHFR showed visible flocculent aggregates in solution at the higher temperatures used for the frataxin partitioning (45 °C) and this was the reason why partitioning was measured at the lower temperature of 25 °C. The reaction mixture was incubated at room temperature and the time dependent changes in concentration of soluble DHFR remaining in solution were measured using UV-visible spectroscopy following the rapid removal of the GroEL beads (brief centrifugation at 1500× *g* for 30 s). The time dependent decline in soluble protein amplitudes were fit to a first order decay curve using Prism curve fitting software (GraphPad Inc.) to generate the macroscopic partitioning rate at each GroEL concentration. These partitioning rates were then plotted as a function of GroEL concentration to obtain the partitioning curve (Supplementary Figure S2).

### 3.5. Differential Scanning Fluorimetry

The effect of the osmolytes on protein’s thermal stability was evaluated through DSF [54,55]. The fluorescent dye Sypro orange (λ_ex_ 300 nm and λ_em_ 470/570 nm) was used as a probe to follow protein unfolding. In all experiments, the heating rates of 1.5 °C/min. Data were analyzed according to a two-state model [56]. The fits to the kinetic unfolding transitions were made using Origin (MicroCal Software, Northampton, MA, USA). The concentration of frataxin used in each experiment was 4 µM. Since dye binding to partially unfolded populations actually induced further time-dependent unfolding for the I154F variant if the dye was added well in advance (e.g., 30 min) of the thermal scan, the dye was added to all wild type and variant species just before starting the thermal scan to ensure that a transition profile could be observed.

### 3.6. Osmolyte Effects on Partitioning

The impact of folding osmolytes on frataxin conformational equilibrium as well as their potential stabilizing effect on folded populations was assessed by measuring the concentration of protein in the soluble phase as a function of time. For this assay, the FXN mutants were incubated with either a weak osmolyte like 4 M glycerol or a strong osmolyte such as 1 M TMAO (Sigma). The mutants were incubated in these osmolyte solutions for 60 min at 45 °C. The UV absorbance spectra of the soluble fractions containing non-partitioned mutants in the presence of the two osmolytes were recorded prior to and after incubation and analyzed for signs of protein aggregation (increase absorbance due to light scattering effects at both 260 nm and 360 nm). A positive outcome results in a constant protein concentration in the presence of immobilized GroEL (*i.e.*, no partitioning) with no apparent aggregation as assessed by light scattering.

## 4. Conclusions

In this study we have used the chaperonin trap platform to evaluate and compare kinetic stabilities of two clinical variants of the frataxin protein, under near physiological conditions. The results presented suggest that the different phenotypes associated with these mutations likely result from their different kinetic instabilities. Furthermore, the chaperonin capture platform may be an effective method for rapidly evaluating potential stabilizers of kinetically metastable states. *In vivo* and *in vitro* assessments of potential osmolyte stabilization indicated that a strong osmolyte such as TMAO drove the more kinetically unstable FXN-p.Ile154Phe mutant towards aggregation while this same osmolyte had very little effect on the aggregation of the FXN-p.Asp122Tyr mutant. Although *in vivo* data indicated that solubility may be increased with osmolytes, the increase in small soluble aggregates could also lead to a false positive result in this instance. Our results support the contention described in recent work by Kelly and colleagues, where general solubility does not equate with the formation of functional protein. The variable effects of strong and weak folding osmolytes may be related to the degree of unfolding and perturbation of internal core structures that are imparted by various missense mutations. Ultimately, this work highlights the potential of using a chaperonin trap system/platform in evaluating subtle kinetic differences in proteins at near physiological temperatures in disease variant missense proteins. From this evaluation, it is entirely possible to rapidly identify small molecules that rescue protein misfolding defects. As methods involving chaperonin capture platforms are advanced, this approach may eventually aid in designing targeted therapeutic approaches specifically for rare orphan disease variants such as the ones encountered in Friedreich’s ataxia heterozygous patients and other misfolding diseases. For example, a superior alternative method to avoid aggregation interferences using a chaperonin partitioning is to immobilize the target protein on a detection surface (e.g., surface plasmon resonance) and flow the chaperonin over that surface to detect binding [17]. In the presence of stabilizing compounds, one would predict that the chaperonin should show diminished binding to a stabilized missense mutant protein. The ability to access the kinetic stability of folding mutants will help determine if one can design global or specific suppressor agents (general or specific protein stabilizers) to ameliorate protein misfolding for a wide range of kinetic stability mutants. Ultimately, these studies indicate that it is beneficial to examine the intrinsic partitioning of missense mutants onto tight binding chaperone surfaces. In the future, it will be interesting to determine if this general *in vitro* partitioning/kinetic GroEL chaperonin trap approach with a kinetically liable mutant platform resembles the *in vivo* interaction tendencies of the same folding mutants with the intracellular proteostatic machinery [57].

## Supplementary Files

Supplementary File 1
