# Supplementary material for: Probing the Kinetic Stabilities of Friedreich’s Ataxia Clinical Variants Using a Solid Phase GroEL Chaperonin Capture Platform
Source: Biomolecules. 2014 Oct 20;4(4):956–79. doi: 10.3390/biom4040956 (PMC4279165; doi:10.3390/biom4040956)
Supplement: Supplementary File 1 [file biomolecules-04-00956-s001.pdf]

Supplementary Information

Figure S1. Partition of frataxin variants onto GroEL beads. (**Top**) SDS/PAGE analysis of the reaction mixture aliquots withdrawn after spin down of the mixture; (**Bottom**) Densitometric analysis of the gels bands, the initial point (before the proteins were incubated at 45 °C) corresponds to 100%. This analysis offers confirmation that the soluble frataxin clinical variant decline when these are incubated with the GroEL coupled beads.

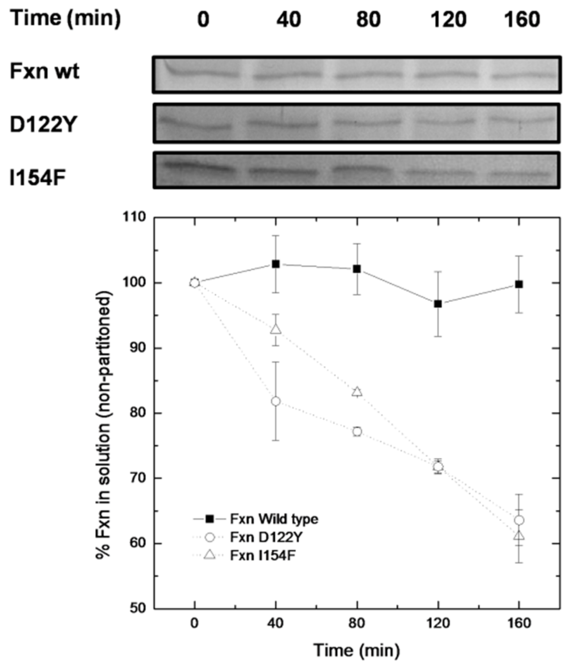

Figure S2. Partitioning rate of DHFR as a function of immobilized GroEL concentration. DHFR is known to partition onto GroEL and this partitioning onto free GroEL in solution has been well characterized [20,21]. DHFR partitioning were rates plotted as a function of immobilized GroEL concentration. The rate follows a hyperbolic function similar to what was previously observed with rhodanese [25] and the frataxin mutants (this paper, Figure 3C). Briefly DHFR was allowed to partition onto the different concentrations of immobilized GroEL beads. The DHFR partitioning rates were calculated by fitting the DHFR partitioning data to a first order decay.

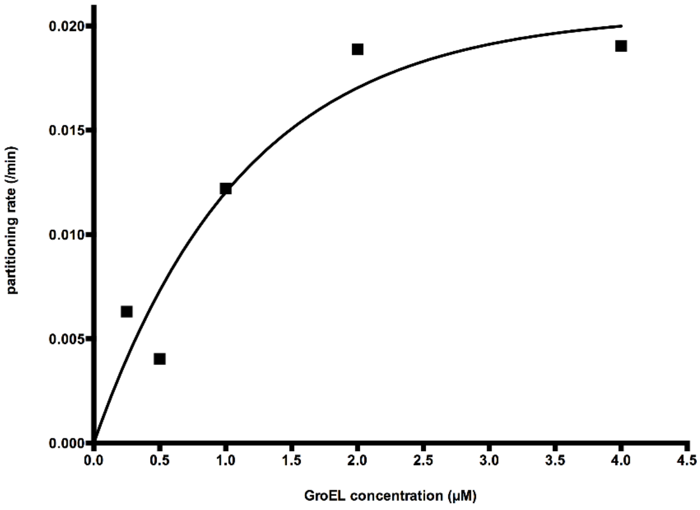

Figure S3. General FXN-p.Asp122Tyr aggregation profiles in solution alone (no osmolytes) (**A**) in the presence of 4 M Glycerol (**B**) or 1 M TMAO (**C**). FXN-p.Asp122Tyr was incubated at 45 °C for 60 min in the absence and presence of the different osmolytes and the UV absorbance spectra at time 0 min and 60 min are represented. As compared to the large aggregation baseline shifts observed in FXN-p.Ile154Phe, the FXN-p.Asp122Tyr spectra does not show large light scattering contributions as assessed by the increase in the entire baseline both in the control without osmolytes (A) or with Glycerol (B) and TMAO (C).

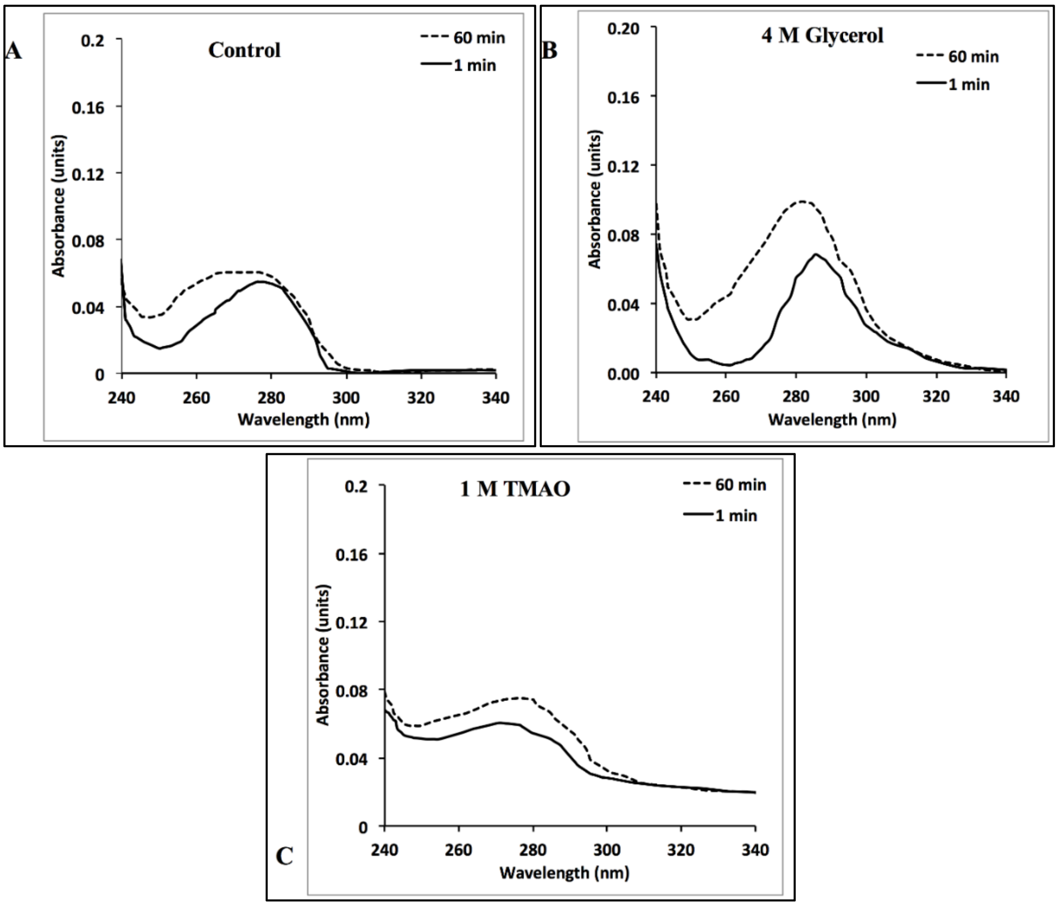

Table S1. Pseudo first order rates calculated by fitting the kinetic profiles presented in Figure 2A,B. The resulting rates were plotted *vs.* increasing GroEL concentrations and this trend is illustrated in Figure 2C.

GroEL Concentration (μM)	Partitioning Rate (s^{-1})	
	**FXN-p.Ile154Phe**	**FXN-p.Asp122Tyr**
0	$0.3 \times 10^{-3} \pm 0.2 \times 10^{-3}$	$0.6 \times 10^{-3} \pm 0.3 \times 10^{-3}$
0.5	$12.3 \times 10^{-3} \pm 0.8 \times 10^{-3}$	$4.5 \times 10^{-3} \pm 0.2 \times 10^{-3}$
1	$12.2 \times 10^{-3} \pm 0.8 \times 10^{-3}$	$5.9 \times 10^{-3} \pm 0.5 \times 10^{-3}$
2	$19.0 \times 10^{-3} \pm 1.4 \times 10^{-3}$	$7.6 \times 10^{-3} \pm 0.4 \times 10^{-3}$
3	$18.9 \times 10^{-3} \pm 2.1 \times 10^{-3}$	$10.5 \times 10^{-3} \pm 0.8 \times 10^{-3}$

© 2014 by the authors; licensee MDPI, Basel, Switzerland. This article is an open access article distributed under the terms and conditions of the Creative Commons Attribution license (http://creativecommons.org/licenses/by/4.0/).
